# Association of Blood Pressure Lowering Intensity With White Matter Network Integrity in Patients With Cerebral Small Vessel Disease

**DOI:** 10.1212/WNL.0000000000201018

**Published:** 2022-10-25

**Authors:** Chris Patrick Pflanz, Marco S. Egle, John T. O'Brien, Robin G. Morris, Thomas R. Barrick, Andrew M. Blamire, Gary A. Ford, Daniel Tozer, Hugh S. Markus, on behalf of the PRESERVE study group

**Affiliations:** From the Stroke Research Group (C.P.P., M.S.E., D.T., H.S.M.), Department of Clinical Neuroscience, University of Cambridge; Department of Psychiatry (J.T.O.B.), University of Cambridge; Kings College Institute of Psychiatry (R.G.M.), Psychology and Neurosciences, London, UK; Molecular and Clinical Science Research Institute (T.R.B.), St George's, University of London, UK; Magnetic Resonance Centre (A.M.B.), Institute of Cellular Medicine, Newcastle University, UK; and Oxford University Hospitals NHS Foundation Trust & University of Oxford (G.A.F.).

## Abstract

**Background and Objectives:**

Diffusion tensor imaging (DTI) networks integrate damage from a variety of pathologic processes in cerebral small vessel disease (SVD) and may be a sensitive marker to detect treatment effects. We determined whether brain network analysis could detect treatment effects in the PRESERVE trial data set, in which intensive vs standard blood pressure (BP) lowering was compared. The primary end point of DTI had not shown treatment differences.

**Methods:**

Participants with lacunar stroke were randomized to standard (systolic 130–140 mm Hg) or intensive (systolic ≤ 125 mm Hg) BP lowering and followed for 2 years with MRI at baseline and at 2 years. Graph theory–based metrics were derived from DTI data to produce a measure of network integrity weighted global efficiency and compared with individual MRI markers of DTI, brain volume, and white matter hyperintensities.

**Results:**

Data were available in 82 subjects: standard n = 40 (mean age 66.3 ± 1.5 years) and intensive n = 42 (mean age 69.6 ± 1.0 years). The mean (SD) systolic BP was reduced by 13(14) and 23(23) mm Hg in the standard and intensive groups, respectively (*p* < 0.001 between groups). Significant differences in diffusion network metrics were found, with improved network integrity (weighted global efficiency, *p* = 0.002) seen with intensive BP lowering. In contrast, there were no significant differences in individual MRI markers including DTI histogram metrics, brain volume, or white matter hyperintensities.

**Discussion:**

Brain network analysis may be a sensitive surrogate marker in trials in SVD. This work suggests that measures of brain network efficiency may be more sensitive to the effects of BP control treatment than conventional DTI metrics.

**Trial Registration Information:**

The trial is registered with the ISRCTN Registry (ISRCTN37694103; doi.org/10.1186/ISRCTN37694103) and the NIHR Clinical Research Network (CRN 10962; public-odp.nihr.ac.uk/QvAJAXZfc/opendoc.htm?document=crncc_users%5Cfind%20a%20clinical%20research%20study.qvw&lang=en-US&host=QVS%40crn-prod-odp-pu&anonymous=true).

**Classification of Evidence:**

This study provides Class II evidence that intensive BP lowering in patients with SVD results in improved brain network function when assessed by DTI-based brain network metrics.

Cerebral small vessel disease (SVD) accounts for 20% of all ischemic strokes and is the most common pathology underlying vascular cognitive impairment and dementia.^1^ Hypertension is a major risk factor for SVD, and lower blood pressure (BP) in midlife is associated with a reduced risk of SVD.^2^ More intensive BP lowering to a target of 120–125 mm Hg systolic has been shown to reduce radiologic SVD in patients without stroke,^3^ but it is uncertain whether similar intensive targets should be applied to patients with symptomatic SVD. Patients with severe SVD have reduced cerebral blood flow and impaired cerebral autoregulation,^4,5^ and excessive BP reduction could lead to hypoperfusion and as a result accelerate white matter (WM) damage and worsen clinical outcomes.^6^

Cognitive testing has been shown to be insensitive to change in patients with SVD over the follow-up durations of 2–3 years used in clinical trials.^7^ This had led to the use of MRI as a surrogate marker to evaluate the treatment efficacy in phase 2 trials in SVD, with a number of MRI biomarkers including WM hyperintensities (WMHs), brain atrophy, and diffusion tensor imaging (DTI) showing sensitivity to detect change over 2–3-year period.^8^ DTI is particularly sensitive to diffuse WM damage in SVD and predicts future dementia risk.^9^ The PRESERVE multicenter randomized clinical trial determined the effect of intensive systolic BP lowering to 125 mm Hg systolic compared with standard BP lowering to 140 mm Hg on WM ultrastructure in SVD, using DTI (change in the WM mean diffusivity [MD] histogram peak height over 2 years) as the primary end point, but did not detect differences between the treatment groups, possibly due to it not reaching the planned sample size.^10^

Recently, structural brain networks, which can be derived using tractography performed on DTI data, have been shown to be disrupted in SVD, with the degree of disruption correlating with cognitive impairment^11,12^ and predicting future dementia risk.^13^ Network disruption mediates the effect of a number of SVD pathologies on cognition, including WMH, lacunes, and DTI WM microstructural alterations.^11^ Thus, network analysis might provide a single measure that integrates the different pathologies in SVD, adding additional information to the degree of WM ultrastructural damage assessed on DTI, and could represent a useful surrogate marker in clinical trials in SVD.

The aim of this, secondary, study was to determine whether network analysis was a more sensitive method to detect treatment effects, in this case the effect of intensive BP lowering, than the primary study end point. As such, the primary question this study was designed to answer was whether network metrics based on DTI-based brain networks could detect treatment effects in the PRESERVE trial data set, in which intensive vs standard BP lowering was compared in participants with established SVD.

## Methods

### Standard Protocol Approvals, Registrations, and Patient Consents

PRESERVE was a 2-year, multicenter, randomized clinical trial comparing intensive vs standard BP treatment options in patients with severe SVD. The full protocol and statistical analysis plan were previously published.^14^ As part of a multimodal 3T MRI acquisition, diffusion data were acquired at baseline and at the 2-year follow-up visit. A detailed description of the PRESERVE clinical trial including the MRI protocol has already been reported.^10,15^ The trial is registered (Clinical Trial registration: ISRCTN37694103^16^, and CRN 10962^17^). Informed participant consent was obtained in line with the Declaration of Helsinki, and the study was approved by the local ethics committee (North London REC3 [REC number 11/LO/0458]).

### Participants and Data Acquired

Participants were included who had a clinical lacunar stroke with an anatomically corresponding lacunar infarct on MRI, in addition to confluent WMH graded as ≥ 2 on the Fazekas scale.^18^ One hundred eleven patients with SVD were randomized to standard (systolic = 130–140 mm Hg) or intensive (systolic ≤125 mm Hg) BP targets, with 56 patients in the standard arm and 55 patients in the intensive arm. Participants were randomized (stratified by center) with random allocation concealed until the intervention was assigned by the local clinician in a 1:1 ratio via a centralized, online system (at Mental Health & Neuroscience Clinical Trials Unit, Kings College London). Due to the nature of the treatment groups, local clinicians were then aware of group allocation. At each clinical checkup (1, 3, 6, 12, 18, and 24 months from the baseline visit), an increase in antihypertensive medication was prescribed if the BP was above the study target (i.e., >125 mm Hg in the standard group and above >140 mm Hg in the intensive group), unless hypotensive symptoms prevented further BP lowering.

Across 6 centers, eight 3-Tesla MR scanners were used (3 Philips Achieva TX and one each of Philips Achieva, Philips Ingenia, Siemens Verio, Siemens Prisma, and Siemens Magnetom Prisma Fit). 3D T1-weighted (T1W), DTI, T2*-weighted (T2*W), and fluid-attenuated inversion recovery (FLAIR) scans were acquired. Rigorous quality control was implemented to ensure standardization of sequence acquisition parameters. In addition to b = 0 s mm^−2^ acquisitions, all DTI acquisitions included 32 equally spaced, noncollinear diffusion gradient directions (b = 1,000 s mm^−2^) to ensure identical angular resolution and noise characteristics. Full details of MR sequences and analysis methods of the MR data have been published,^15^ and brief details of the relevant sequences are shown in Table 1. All MR analysis was performed centrally and blinded to subject identity and treatment arm.

**Table 1:**
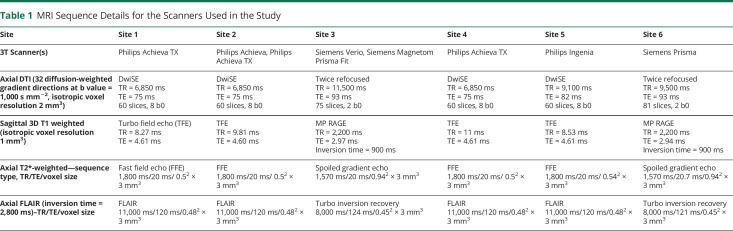
MRI Sequence Details for the Scanners Used in the Study

### MRI Data Analysis and Construction of Brain Networks

WMHs were defined as areas of increased signal on FLAIR images, segmented by a single trained rater using a semiautomated contouring technique in Jim version 7.0_5 (Xinapse Systems Limited^19^). A WMH lesion load score was calculated as the percentage of WMH lesion volume against whole-brain volume.

Lacunes were defined as CSF-filled cavities at least 3 mm in diameter using a combination of T1W, T2*W, and FLAIR scans. Additional features such as T2-hyperintense rims, shape, and location were also considered to differentiate lacunes from similar imaging features.

T1W scans were intensity nonuniformity corrected and segmented into gray matter (GM), WM, and CSF tissue probability maps (TPMs) using SPM12b.^20^ Brain volume was calculated from the GM and WM TPMs. These volumes were normalized by applying SIENAX^21^ to the T1W scans giving a scaling factor. The brain volumes were multiplied by this scaling factor to provide normalized brain volumes.

Diffusion data were preprocessed to correct for geometric distortions and eddy currents and a diffusion tensor model fit at each voxel using the Oxford Centre for Functional MRI of the Brain Software Library (FSL)^22^ to produce fractional anisotropy (FA) and MD maps. T1W images were brain extracted using the FSL brain extraction tool (BET)^23^ and coregistered into MNI space using Advanced Normalization Tools.^24^ Non–diffusion-weighted b = 0 s mm^−2^ (B0) images were registered onto the brain-extracted T1W images using the FSL tool flirt. This transform was used to generate WM masks for the diffusion data. Histogram analysis was performed on FA and MD maps in WM. Normalized histograms with 1,000 bins (FA range 0–1, bin width 0.001; MD range 0–4 mm^2^ s^−1^ × 10^−3^, bin width 0.004 mm^2^ s^−1^ × 10^−3^) were computed, and the median, peak height, and peak value were extracted from these for both FA and MD. In addition, 90 seed regions were defined from the Desikan-Killiany parcellation of the cerebral cortex and subcortical nuclei^25^ using the automatic-anatomic labeling (AAL) template without the cerebellum and brainstem in Montreal Neurological Institute (MNI) space. Inverse warps/matrices from the registration of the B0 to T1W space and then from T1W space to MNI space were used to transform the AAL seed masks into native diffusion space for each subject and time point.

Deterministic whole-brain tractography was run on the principal eigenvectors of the diffusion tensor using MRtrix.^26^ In brief, this algorithm fits a diffusion tensor to the local (trilinear-interpolated) diffusion data at each streamline step, and the streamline trajectory is then determined as the principal eigenvector of that tensor.^27^ The following additional settings were used: step size = 0.5 mm; maximum angle theta between successive steps: 45°; minimum length of any track: 20 mm; maximum length of any track: 250 mm; tensor FA cutoff threshold for terminating tracks: 0.15; integration method used to fit streamlines to the tensor field: 4th-order Runge-Kutta integration; grid size: 4; and streamlines terminating in their region of origin were removed. From the whole-brain deterministic tractogram, connections were generated between each pair of seed masks. Two brain regions A and B were considered to be connected with each other if one or more streamlines terminating in region A also terminated in region B. Streamlines were seeded within voxel on an evenly spaced super-resolution grid (0.5 mm^3^), due to this the strength of connectivity between each pair of seeds was calculated as the streamline count between regions adjusted for the length of the streamline in mm. This was performed by scaling each contribution to the connectome edge by the inverse of the streamline length due to the presence of multiple seeding points in each streamline.

**Figure 1:**
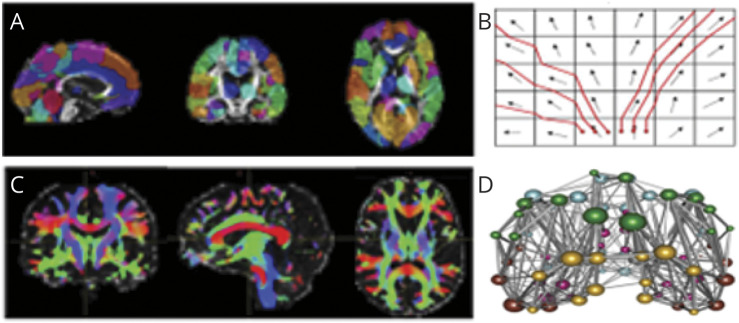
Tractography-Based Image Processing Pipeline Connectome-based network analysis workflow in the MRI branch of the PRESERVE clinical trial investigating longitudinal effects on networks metrics at baseline and the follow-up visit at 2 years. (A) AAL atlas overlaid on the MRI. (B) Streamline construction. (C) Whole-brain tractography. (D) Reconstructed connectome used to calculate the measures such as global efficiency. AAL = automatic-anatomic labeling.

On the basis of this strength of connectivity, the connectome was reconstructed as an undirected graph, and the adjacency matrix was created as a symmetric matrix where each element represents the strength of connectivity between each pair of brain regions. From the adjacency matrix, graph-theoretical network metrics were calculated using the brain graph package^28^ and igraph package available in R.^29^ Given that there are a large number of network metrics, which are often highly correlated, we chose to focus on weighted average global efficiency and weighted averaged local efficiency. In brief, global efficiency indicates how well connected all nodes of the brain network are relative to an idealized brain network where each and every node is connected. Local efficiency tells us how resistant the brain network is against failure in information processing on a small scale. The connectome-based network analysis workflow, described above, is summarized in (Figure 1).

### Data Analysis

**Figure 2:**
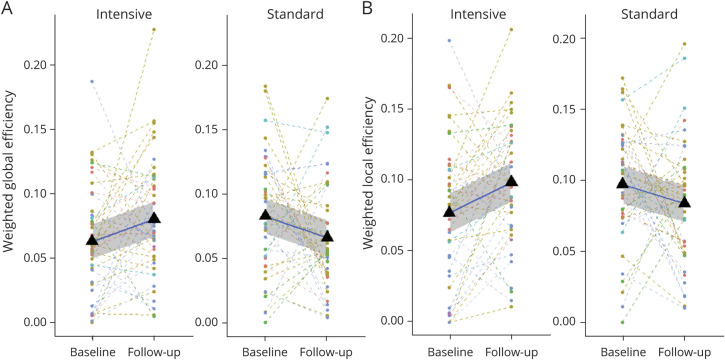
Change in Global and Local Efficiency Between Time Points Longitudinal plots showing the change in weighted global efficiency (A) and weighted local efficiency (B) in response to antihypertensive therapy in the intention-to-treat group showing the differences in behaviors between the 2 treatment groups. Pairs of dots relate to individual participants; they are colored by study site. The triangles and thick line show the mean behavior for the group, and the shaded area around the mean line is the standard error.

The primary analysis was the intention-to-treat group. In addition, a secondary per-protocol analysis was performed that included only patients reaching the BP target as previously defined.^10^ Statistical analysis was performed using R (version 3.2.3) and the statistical models package (stats models) as implemented in python.^30^ During the exploratory data analysis performed on the diffusion network metrics, we noticed a large amount of outliers both longitudinally and cross-sectionally exceeding the 3-sigma rule (see also the dispersion plots shown in Figure 2). We therefore decided to use permutational tests to assess the effects against bias and because permutational analysis of covariance models are robust against both outliers and violation of the assumption of normality.

Permutational repeated-measures analysis of covariance (ANCOVA) with study site as a covariate and treatment group (standard vs intensive) as an explanatory factor was used to test for significant group effects and group-by-time-point interactions. Within this framework, main effects of the time point correspond to longitudinal effects on diffusion metrics irrespective of the group, main effects of the treatment group correspond to group differences irrespective of the time point, and time-point-by-treatment-group interactions correspond to the longitudinal effects of the therapeutic intervention on diffusion network metrics. This model has greater statistical power to detect any effect when compared with analysis of covariance with baseline as a covariate or analysis of variance performed on the differences between baseline and follow-up (the so-called delta values). To assess whether any associations were merely due to network metrics acting as a nonspecific measure of SVD severity, we also covaried for WMH volume as a measure of SVD severity.

### Data Availability

The data supporting the findings of the study may be obtained from the corresponding author on reasonable request from a qualified researcher.

## Results

Recruitment took place from February 29, 2012, to October 10, 2015; follow-up was completed on November 1, 2017. Participants were recruited from 6 secondary care stroke services sites across the United Kingdom. One subject did not meet the MRI criteria on baseline central MRI review and was withdrawn. Three died during follow-up, 1 developed other serious illness and could not continue, 6 withdrew consent, and 2 were lost to follow-up. Baseline MRI was not performed in 2, and follow-up MRI was not performed in 6 participants. Therefore, 90 participants remained with baseline and follow-up MRI scans. After excluding scans of inadequate quality for DTI analysis, 40 patients in the standard arm and 42 patients in the intensive arm were included in the graph-theoretical data analysis, and assessment of image quality was made blinded to subject number and treatment group. A subject flow diagram representing the above can be seen in^10^ Figure 1.

**Figure 3:**
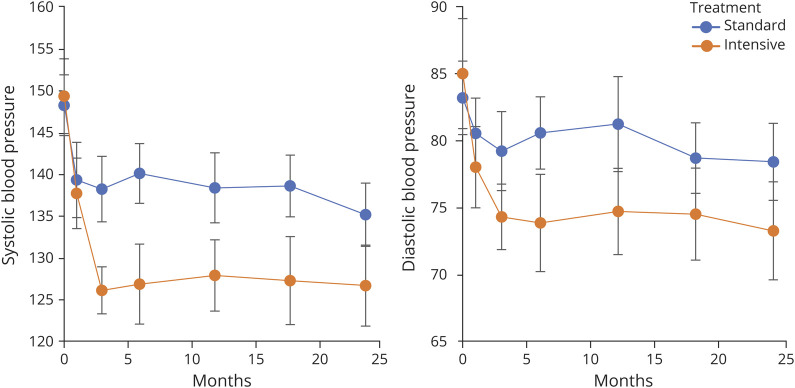
Blood Pressure Change Across the Study Period Reductions in blood pressure over the course of the clinical trial for the standard and the intensive treatment groups. The blue lines show the standard treatment group, and the orange lines show the intensive treatment group. Error bars show the 95% CI. Panel A shows the systolic blood pressure, and panel B the diastolic.

There was no difference in demographics between those included (n = 82) and excluded (n = 8) in the study in demographics, cognition, or MRI metrics (age: 68 ± 12 vs 66 ± 6 years, *p* = 0.33, NART: 116 ± 9 vs 116 ± 12, *p* = 0.96, global cognition: −0.78 ± 0.99 vs −0.49 ± 0.61, *p* = 0.42, systolic BP, 147 ± 21 vs 155 ± 16, *p* = 0.22, CMB: 4 ± 8 vs 3 ± 5, *p* = 00.59, lacunes 4 ± 3 vs 4 ± 8, p0.93, lesion load: 3.4% ± 2.3 vs 3.5% ± 2.3, *p* = 0.87, normalized brain volume 1,333 mL ± 185 vs 1,406 mL ± 137, *p* = 0.17). In the 82 patients whose data included in this analysis, there was no difference in baseline BP (Table 2). Target BP difference was achieved by 3 months (intensive 127 mm Hg and standard 140 mm Hg) and maintained for 2 years (as shown in Figure 3). The mean (SD) systolic BP was reduced by −13 (14) and −23 (23) mm Hg in the standard/intensive groups, respectively (difference between groups *p* < 0.001). The number reaching BP targets at 3 months and included in the per-protocol analysis were standard arm 35/40 and intensive arm 28/42.

**Table 2:**
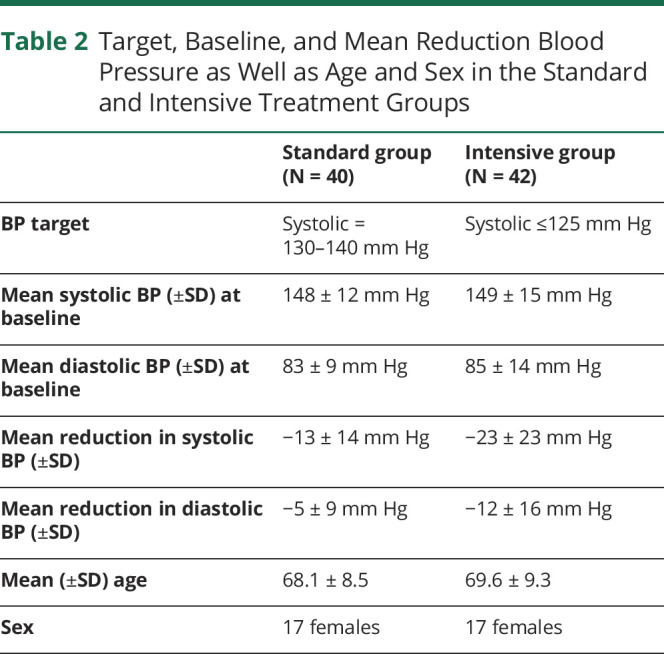
Target, Baseline, and Mean Reduction Blood Pressure as Well as Age and Sex in the Standard and Intensive Treatment Groups

Permutational repeated-measures ANCOVA with study site as a covariate showed that intensive BP lowering was associated with no progression of network disruption over the 2-year follow-up and even a possible improvement, whereas standard BP lowering was associated with a decline in network metrics (Table 3): weighted global efficiency (*p* = 0.002, Figure 2A) and weighted local efficiency (*p* = 0.002, Figure 2B), where a reduction in the efficiency was seen over 2 years for the standard treatment regime with a corresponding increase in the intensive group. Figure 2 shows the behavior of the individual participants (color coded by site) and the mean change as the thick line surrounded by the standard error (shaded area).

**Table 3:**
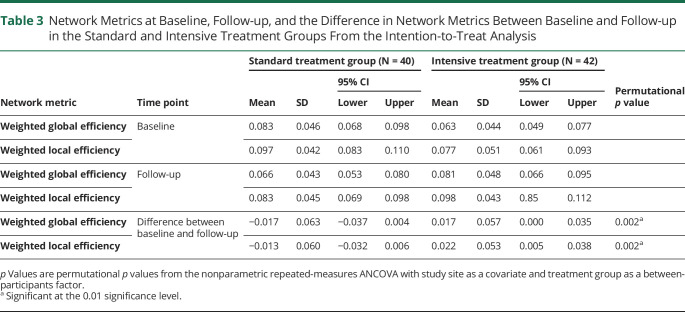
Network Metrics at Baseline, Follow-up, and the Difference in Network Metrics Between Baseline and Follow-up in the Standard and Intensive Treatment Groups From the Intention-to-Treat Analysis

To assess whether the associations seen in Table 3 were merely due to network metrics acting as a nonspecific measure of SVD severity, we also covaried for WMH lesion volume as a measure of SVD severity, entering both the magnitude of lesion load at baseline (intercept) and the longitudinal change (slope) of the lesion load as covariates into the repeated-measures model. The directionality and effects of treatment regimen on the network metrics remained after this analysis: weighted global efficiency (*p* = 0.007) and weighted local efficiency (*p* = 0.005).

In contrast to the significant difference between treatment groups on network analysis, we found no difference when using conventional DTI parameters in a histogram-based analysis. Permutational repeated-measures ANCOVA with study site as a covariate did not show any significant effects of treatment regimen for the following histogram metrics in the normal-appearing WM: FA peak height (permutational *p* = 0.99), MD peak height (*p* = 0.99), FA peak location (*p* = 0.244), MD peak location (*p* = 0.99), median FA (*p* = 0.071), and median MD (*p* = 0.99).

Similarly, no significant overall effects of treatment regimen were found for normalized brain volumes, lesion volumes, and microbleeds, including the following: normalized whole-brain volume (*p* = 0.076), normalized GM volume (*p* = 0.941), normalized WM volume (*p* = 0.222), WMH lesion load (*p* = 0.415), lacunes (*p* = 0.863), and microbleeds (*p* = 0.278).

On per-protocol analysis only including participants reaching their BP target, there were similar results. There were significant effects of treatment regimen (i.e., standard vs intensive intention-to-treat groups) for weighted local efficiency (*p* = 0.016), but not weighted global efficiency (*p* = 0.055) (Table 4) with the same directionality of changes seen as in the whole-group analysis.

**Table 4:**
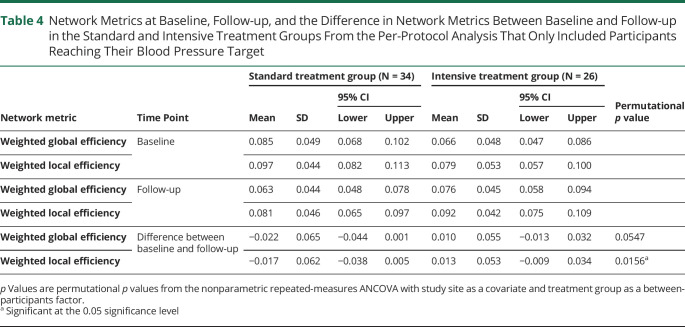
Network Metrics at Baseline, Follow-up, and the Difference in Network Metrics Between Baseline and Follow-up in the Standard and Intensive Treatment Groups From the Per-Protocol Analysis That Only Included Participants Reaching Their Blood Pressure Target

### Classification of Evidence

The primary question this study was designed to answer was whether network metrics based on DTI-based brain networks could detect treatment effects in the PRESERVE trial data set, in which intensive vs standard BP lowering was compared in participants with established SVD. This study provides Class II evidence that intensive BP lowering in patients with SVD results in improved brain network function when assessed by DTI-based brain network metrics. It used a well-designed randomized controlled trial data set but was a post hoc analysis and not the primary end point. It is found that intensive BP lowering results in improved brain network function when measured with global and local efficiency.

## Discussion

In this analysis of the PRESERVE data set, using diffusion network metrics as the outcome measure, intensive BP reduction of systolic BP to 125 mm Hg was associated with reduced WM damage in patients with severe SVD. Our results suggest that network metrics may be a more sensitive surrogate marker for multicenter clinical trials in patients with SVD than conventional DTI analyses. It also suggests that intensive BP lowering is beneficial even in patients with severe SVD, extending data demonstrating its efficacy in primary prevention and in patients with milder SVD.

Network disruption has been shown to mediate the effect of a number of SVD pathologies including WMH, diffuse WM damage on DTI, lacunes, and cerebral microbleeds on cognitive impairment.^11,12^ It may therefore provide a more comprehensive measure of SVD pathology than a simple DTI metric, and this may explain its greater sensitivity to detecting a treatment effect in the PRESERVE data set. In this analysis, it provided greater sensitivity than WM MD peak height, a metric derived from DTI histogram analysis, which was the primary end point of the PRESERVE trial.^10^

Our results show that both global and local efficiency showed progressive deterioration in this standard BP arm, whereas they showed no deterioration and even a possible improvement in the intensive BP arm. This is consistent with intensive BP lowering leading to an improvement in network integrity. Why intensive BP lowering would lead to an improvement, rather than stabilization, of network metrics is unclear. One possible explanation is that the reduction in BP reduces inflammation, which has been shown to be present in SVD,^31,32^ resulting in less cell swelling and tissue infiltration, and this could lead to an overall increase in network strength as brain regions would appear to be more connected, rather than a genuine increase in connectivity. It has previously been hypothesized that antihypertensive therapy may reduce ischemia-induced neuroinflammation in patients with stroke.^33^ Alternatively, it is possible that the changes seen are due to remyelination and repair of the axons as inflammatory episodes are reduced or stopped. This would indicate a genuine increase in tissue health leading to greater connectivity.

This article provides further support for the beneficial effect of intensive BP lowering in patients with SVD. The SPRINT trial, in the setting of primary prevention, showed that intensive BP lowering to 125 mm Hg systolic was associated with a reduction in both cardiovascular events^34^ and a reduction in the combined end point of dementia and mild cognitive impairment.^35^ An MRI substudy of SPRINT demonstrated that intensive BP lowering was also associated with reduced progression of radiologic SVD, as determined by WMH.^3^ The SPS3 trial, in patients with lacunar stroke, showed no significant difference in the recurrent stroke rate with intensive BP lowering, but the researchers suggested a possible trend in stroke reduction.^36^ Therefore, increasing evidence suggests that more intensive BP lowering may reduce cardiovascular events and possibly also dementia. However, there has been concern that in patients with severe SVD, in whom reduced cerebral blood flow and impaired cerebral autoregulation have been found, this could result in a drop in cerebral blood flow, which in itself could result in increased WM damage and impaired cognition. This analysis provides support for intensive BP lowering, even in patients with severe SVD.

Cognitive end points have been shown to be insensitive to change in clinical trials in SVD. This has led to increasing interest in the use of MRI surrogate markers to assess the efficacy in phase 2 trials before large phase 3 studies with dementia as the clinical end point. A number of MRI markers have been suggested as useful surrogates including WMH, WM ultrastructural change on DTI, and brain volume.^8^ Our study suggests that a measure that integrates a number of the underlying SVD radiologic pathologies, which network metrics do, may be a sensitive marker. In particular, network analysis was more powerful than conventional DTI markers.

Our study has a number of strengths. It is one of the few studies in SVD to use a randomized controlled trial methodology to evaluate a surrogate marker. We found consistent results across both the intention-to-treat and per-protocol analyses. All MR analyses were preformed blinded to treatment allocation. However, it also has limitations. This was a secondary analysis of a clinical trial data set for which the primary end point was a simple DTI histogram metric. The original sample size of the PRESERVE trial was 180, and only 111 patients were recruited to the MRI arm, the smaller sample size, and exclusions do reduce the power of the study. A post hoc power calculation indicates that the power of the study ranges between 58% and 73% for the analyses and parameters. The per-protocol analysis produced weaker results than the intention-to-treat analysis; this is most likely to be caused by the reduced number of participants in the per-protocol analysis. A number of participants go the ‘wrong way’ in both groups; which may be for a number of reasons. This may just be natural variation in the data set, which would suggest that although network measures can detect treatment effects across groups, they are less useful in detecting change in individual participants.

In conclusion, intensive systolic BP lowering to a target of 125 mm Hg was associated with less progression of WM damage as assessed by network metrics. This supports the use of intensive BP targets even in patients with severe SVD. Diffusion network metrics may be a more sensitive surrogate marker for multicenter clinical trials in patients with SVD than conventional DTI analyses and other MRI measures.

## Glossary

AAL: automatic-anatomic labeling
BP: blood pressure
DTI: diffusion tensor imaging
FA: fractional anisotropy
FLAIR: fluid-attenuated inversion recovery
FSL: Oxford Centre for Functional MRI of the Brain Software Library
GM: gray matter
MD: mean diffusivity
MNI: Montreal Neurologic Institute
SVD: small vessel disease
T1W: T1 weighted
T2*W: T2* weighted
TPMs: tissue probability maps
WM: white matter
WMH: WM hyperintensity

## References

[1] Pantoni L. Cerebral small vessel disease: from pathogenesis and clinical characteristics to therapeutic challenges. Lancet Neurol. 2010;9(7):689-701. 20610345 10.1016/S1474-4422(10)70104-6

[2] Abell JG, Kivimäki M, Dugravot A, et al. Association between systolic blood pressure and dementia in the Whitehall II cohort study: role of age, duration, and threshold used to define hypertension. Eur Heart J. 2018;39(33):3119-3125. 29901708 10.1093/eurheartj/ehy288 PMC6122131

[3] SPRINT MIND Investigators for the SPRINT Research Group, Pajewski NM, Chelune G, Chelune G, et al. Association of intensive vs standard blood pressure control with cerebral white matter lesions. JAMA. 2019;322(6):524-534. 31408137 10.1001/jama.2019.10551 PMC6692679

[4] Bakker SL, de Leeuw FE, de Groot JC, Hofman A, Koudstaal PJ, Breteler MM. Cerebral vasomotor reactivity and cerebral white matter lesions in the elderly. Neurology. 1999;52(3):578-583. 10025791 10.1212/wnl.52.3.578

[5] Immink RV, van Montfrans GA, Stam J, Karemaker JM, Diamant M, van Lieshout JJ. Dynamic cerebral autoregulation in acute lacunar and middle cerebral artery territory ischemic stroke. Stroke. 2005;36(12):2595-2600. 16254228 10.1161/01.STR.0000189624.06836.03

[6] Birns J, Markus H, Kalra L. Blood pressure reduction for vascular risk: is there a price to be paid? Stroke. 2005;36(6):1308-1313. 15860749 10.1161/01.STR.0000165901.38039.5f

[7] Pearce LA, McClure LA, Anderson DC, et al, SPS3 Investigators. Effects of long-term blood pressure lowering and dual antiplatelet treatment on cognitive function in patients with recent lacunar stroke: a secondary analysis from the SPS3 randomised trial. Lancet Neurol. 2014;13(12):1177-1185. 25453457 10.1016/S1474-4422(14)70224-8 PMC4284947

[8] Benjamin P, Zeestraten E, Lambert C, et al. Progression of MRI markers in cerebral small vessel disease: sample size considerations for clinical trials. J Cereb Blood Flow Metab. 2016;36(1):228-240. 26036939 10.1038/jcbfm.2015.113 PMC4758545

[9] Zeestraten EA, Lawrence AJ, Lambert C, et al. Change in multimodal MRI markers predicts dementia risk in cerebral small vessel disease. Neurology. 2017;89(18):1869-1876. 28978655 10.1212/WNL.0000000000004594 PMC5664300

[10] Markus HS, Egle M, Croall ID, et al, PRESERVE Study Team. PRESERVE: randomized trial of intensive versus standard blood pressure control in small vessel disease. Stroke. 2021;52(8):2484-2493. 34044580 10.1161/STROKEAHA.120.032054

[11] Lawrence AJ, Chung AW, Morris RG, Markus HS, Barrick TR. Structural network efficiency is associated with cognitive impairment in small-vessel disease. Neurology. 2014;83(4):304-311. 24951477 10.1212/WNL.0000000000000612 PMC4115608

[12] Tuladhar AM, Tay J, van Leijsen E, et al. Structural network changes in cerebral small vessel disease. J Neurol Neurosurg Psychiatry. 2020;91(2):196-203. 31744851 10.1136/jnnp-2019-321767

[13] Lawrence AJ, Zeestraten EA, Benjamin P, et al. Longitudinal decline in structural networks predicts dementia in cerebral small vessel disease. Neurology. 2018;90(21):e1898-e1910. 29695593 10.1212/WNL.0000000000005551 PMC5962914

[14] PRESERVE trial protocol. Accessed April 27, 2022 neurology.cam.ac.uk/wp-content/uploads/2014/04/PRESERVE-Protocol-Version-5-13-December-2013.pdf

[15] Croall ID, Lohner V, Moynihan B, et al. Using DTI to assess white matter microstructure in cerebral small vessel disease (SVD) in multicentre studies. Clin Sci (Lond). 2017;131(12):1361-1373. 28487471 10.1042/CS20170146 PMC5461938

[16] How intensively should we treat blood PRESsure in established cERebral small VEssel disease? ISRCTN registry. doi: 10.1186/ISRCTN37694103. Accessed April 27, 2022.

[17] ISRCTN Registry. Accessed April 27, 2022. 10.1186/ISRCTN37694103.

[18] NIHR CRN Public Search. Accessed April 29, 2022. public-odp.nihr.ac.uk/QvAJAXZfc/opendoc.htm?document=crncc_users%5Cfind%20a%20clinical%20research%20study.qvw&lang=en-US&host=QVS%40crn-prod-odp-pu&anonymous=true.

[19] Fazekas F, Chawluk JB, Alavi A, Hurtig HI, Zimmerman RA. MR signal abnormalities at 1.5 T in Alzheimer's dementia and normal aging. AJR Am J Roentgenol. 1987;149(2):351-356. 3496763 10.2214/ajr.149.2.351

[20] Jim 9. Xinapse Systems. Accessed April 27, 2022. xinapse.com/.

[21] SPM12. Accessed April 27, 2022. fil.ion.ucl.ac.uk/spm/software/spm12/.

[22] Smith SM, Zhang Y, Jenkinson M, et al. Accurate, robust and automated longitudinal and cross-sectional brain change analysis. NeuroImage. 2002;17(1):479-489. 12482100 10.1006/nimg.2002.1040

[23] Jenkinson M, Beckmann CF, Behrens TEJ, Woolrich MW, Smith SM. NeuroImage. 2012;62(2):782-790. 21979382 10.1016/j.neuroimage.2011.09.015

[24] Smith SM. Fast robust automated brain extraction. Hum Brain Mapp. 2002;17(3):143-155. 12391568 10.1002/hbm.10062 PMC6871816

[25] Avants BB, Tustison NJ, Stauffer M, Song G, Wu B, Gee JC. The Insight ToolKit image registration framework. Front Neuroinform. 2014;8:44. 24817849 10.3389/fninf.2014.00044 PMC4009425

[26] Desikan RS, Ségonne F, Fischl B, et al. An automated labeling system for subdividing the human cerebral cortex on MRI scans into gyral based regions of interest. Neuroimage. 2006;31(3):968-980. 16530430 10.1016/j.neuroimage.2006.01.021

[27] Tournier J-D, Calamante F, Connelly A. MRtrix: diffusion tractography in crossing fiber regions. Int J Imaging Syst Technol. 2012;22:53-66.

[28] Basser PJ, Pajevic S, Pierpaoli C, Duda J, Aldroubi A. In vivo fiber tractography using DT-MRI data. Magn Reson Med. 2000;44(4):625-632. 11025519 10.1002/1522-2594(200010)44:4<625::aid-mrm17>3.0.co;2-o

[29] Watson CG, Stopp C, Newburger JW, Rivkin MJ. Graph theory analysis of cortical thickness networks in adolescents with d-transposition of the great arteries. Brain Behav. 2018;8(2):e00834. 29484251 10.1002/brb3.834 PMC5822582

[30] R Core Team. R: A Language and Environment for Statistical Computing. R Foundation for Statistical Computing; 2012. Accessed April 27, 2022. R-project.org/.

[31] Seabold S, Perktold J. Statsmodels: econometric and statistical modeling with python. Proceedings of the 9th Python in Science Conference 2010:92-95.

[32] Low A, Mak E, Malpetti M, et al. In vivo neuroinflammation and cerebral small vessel disease in mild cognitive impairment and Alzheimer's disease. J Neurol Neurosurg Psychiatry. 2020;92:45-52. 10.1136/jnnp-2020-323894 PMC7803899 32917821

[33] Walsh J, Tozer DJ, Sari H, et al. Microglial activation and blood-brain barrier permeability in cerebral small vessel disease. Brain. 2021;144(5):1361-1371. 34000009 10.1093/brain/awab003 PMC8874873

[34] Sörös P, Whitehead S, Spence JD, Hachinski V. Antihypertensive treatment can prevent stroke and cognitive decline. Nat Rev Neurol. 2013;9(3):174-178. 23247612 10.1038/nrneurol.2012.255

[35] SPRINT Research Group, Williamson JD, Snyder JK, Snyder JK, et al. A randomized trial of intensive versus standard blood-pressure control. N Engl J Med. 2015;373(22):2103-2116. 26551272 10.1056/NEJMoa1511939 PMC4689591

[36] SPRINT MIND Investigators for the SPRINT Research Group, Williamson JD, Pajewski NM, Bryan RN, et al. Effect of intensive vs standard blood pressure control on probable dementia: a randomized clinical trial. JAMA. 2019;321(6):553-561. 30688979 10.1001/jama.2018.21442 PMC6439590

[37] SPS3 Study Group, Benavente OR, Coffey CS, Hart RG, et al. Blood-pressure targets in patients with recent lacunar stroke: the SPS3 randomised trial. Lancet. 2013;382(9891):507-515. 23726159 10.1016/S0140-6736(13)60852-1 PMC3979302

